# Re-Emerging Vaccine-Preventable Diseases in War-Affected Peoples of the Eastern Mediterranean Region—An Update

**DOI:** 10.3389/fpubh.2017.00283

**Published:** 2017-10-25

**Authors:** Rasha Raslan, Skye El Sayegh, Sana Chams, Nour Chams, Angelo Leone, Inaya Hajj Hussein

**Affiliations:** ^1^Faculty of Medicine, American University of Beirut, Beirut, Lebanon; ^2^Department of Internal Medicine, Wayne State University School of Medicine, Rochester, MI, United States; ^3^Department of Experimental and Clinical Neurosciences, University of Palermo, Palermo, Italy; ^4^Department of Biomedical Sciences, Oakland University William Beaumont School of Medicine, Rochester, MI, United States

**Keywords:** re-emerging infections, vaccine-preventable diseases, refugees, poliomyelitis, measles, cholera

## Abstract

For the past few decades, the Eastern Mediterranean Region has been one area of the world profoundly shaped by war and political instability. On-going conflict and destruction have left the region struggling with innumerable health concerns that have claimed the lives of many. Wars, and the chaos they leave behind, often provide the optimal conditions for the growth and re-emergence of communicable diseases. In this article, we highlight a few of the major re-emerging vaccine preventable diseases in four countries of the Eastern Mediterranean Region that are currently affected by war leading to a migration crisis: Iraq, South Sudan, Syria, and Yemen. We will also describe the impact these infections have had on patients, societies, and national health care services. This article also describes the efforts, both local and international, which have been made to address these crises, as well as future endeavors that can be done to contain and control further devastation left by these diseases.

## Introduction

In 1963, Dr. T. Aidan Cockburn, in his book called *The Evolution and Eradication of Infectious Diseases*, stated that “*it seems reasonable to anticipate that within some measurable time* … *all the major infections will have disappeared*” (1). Cockburn was writing in an era when it was believed that infectious diseases would soon be a thing of the past. During the 1950s and 1960s, developing countries saw a tremendous increase in vaccination campaigns against polio, smallpox, measles, and many other common infections. However, as new and old diseases continued to emerge, it soon became apparent that this initial optimism was misguided (2).

A newly emerging disease is one that has not been described or identified before. Re-emerging infectious diseases, on the other hand, are diseases that although were once eradicated, have later resurfaced to pose a significant health problem once again (3). Many of these infections were eradicated with the advent of effective vaccines. In fact, the world has witnessed an upward trend in the rates of vaccination coverage from 74% in 2000 to 85% just 10 years later (4). Despite these strides, in the twenty-first century, infectious diseases continue to be one of the leading causes of death in developing countries (5).

There have been numerous factors attributed to disease re-emergence. These include microbial adaptation and change, increased human susceptibility to infection, changing environments, evolving human demographics and behavior, economic development, international travel, antimicrobial resistance, breakdown in public health measures, increased poverty and social inequality, and on-going war and famine (6). This is particularly true in vulnerable populations such as refugees and displaced peoples who are faced with innumerable obstacles in obtaining adequate health care.

**Figure 1:**
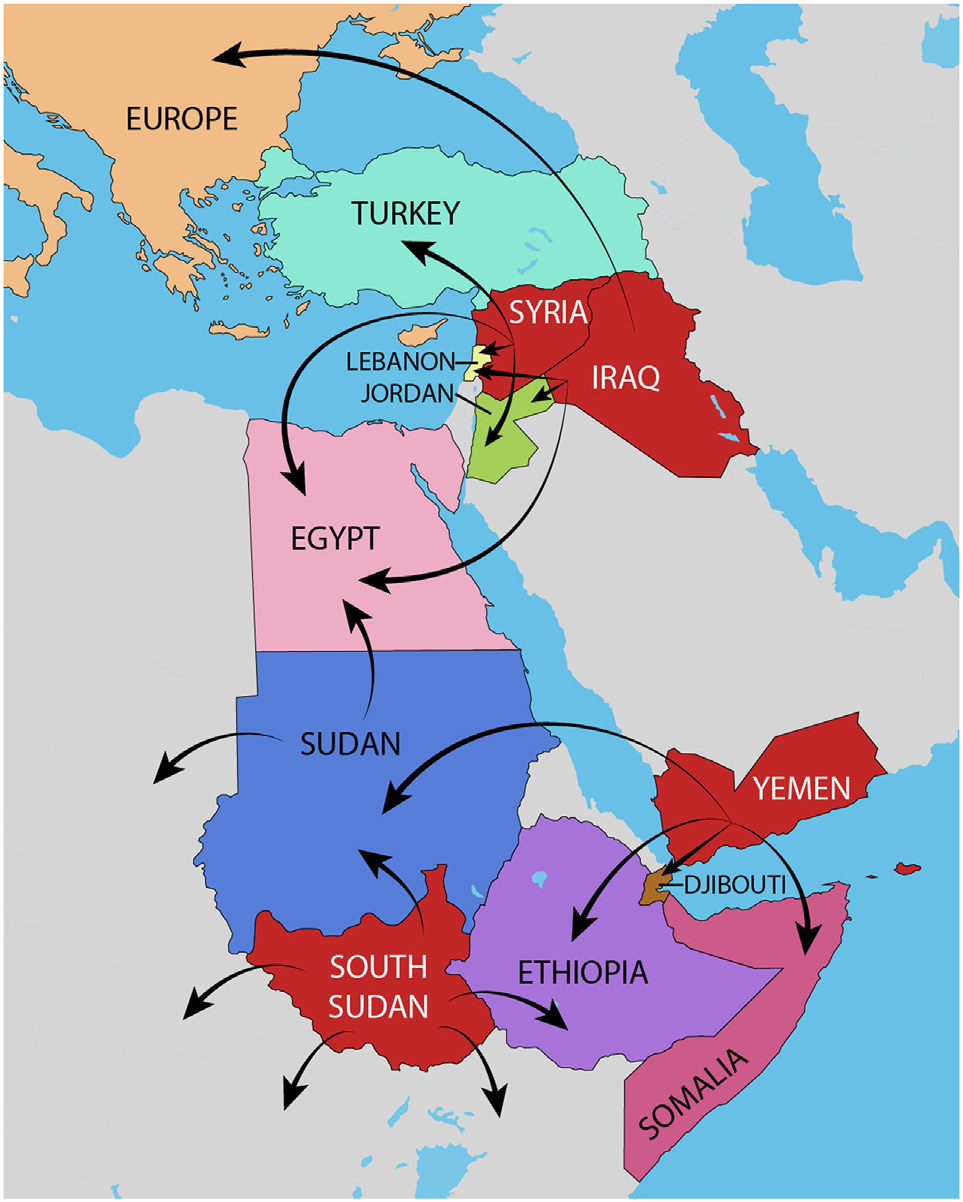
Map of the Eastern Mediterranean Region. The four countries of interest, Iraq, South Sudan, Syria, and Yemen are depicted in red. The main migratory routes of refugees from these countries to neighboring countries are mapped with arrows.

The World Health Organization (WHO) Eastern Mediterranean Region (EMR) is one area of the world that is currently undergoing major political and economic upheaval. Of the 22 countries that comprise the region, Iraq, South Sudan, Syria, and Yemen, are four countries currently undergoing continuous conflict leading to mass displacement. These countries, in addition to the migratory routes undertaken by refugees to neighboring countries, are mapped in Figure 1. Along with the political disturbances, a major health crisis has been brewing for some time now with the rise of once-eradicated communicable diseases. It seems that a future without infectious diseases is a long way away.

In this review, we will highlight the delicate interplay between war, displacement, and the re-emergence of once eradicated vaccine-preventable diseases (VPDs), namely cholera, measles, and polio, as illustrated in Table 1. Disease re-emergence will be described in the context of the aforementioned countries’ political, economic, and health care scenes. Finally, we will provide several recommendations on how to avert the rise of these infections, tackling various social, political, and financial barriers.

**Table 1:** Summary of three major re-emerging vaccine-preventable diseases in the Eastern Mediterranean Region.

Human disease	Infectious agent	Mode of transmission	Symptoms	Treatment	Available vaccine	Re-emerging EMR countries
Poliomyelitis^a^	Polio virus	Fecal–oral	Asymptomatic, may affect the CNS and cause paralysis	Supportive	*Salk Vaccine*—IPV: inactivated polio vaccine *Sabin Vaccine*—OPV: oral polio vaccine	Syria, Iraq
Measles^b^	Measles virus	Direct contact with infectious droplet or by airborne spread	Maculopapular rash, fever, cough, coryza, conjunctivitis, koplik spots	Supportive	Either alone or as part of a combination (e.g., MMR, MMRV)	Iraq, South Sudan, Syria, Yemen
Cholera^c^	*Vibrio cholerae* bacteria	Fecal–oral through contaminated food and water	Profuse watery diarrhea (“rice-water” diarrhea), symptoms of dehydration	Rehydration therapy, antibiotics for severe illness	Two oral killed vaccines: Dukoral and Shanchol (Shantha Biotechnics-Sanofi Pasteur)	South Sudan, Yemen

*^a^Mehndiratta et al. (76)*.

*^b^Naim (77)*.

*^c^Harris et al. (78)*.

## Overview of the Re-Emerging Vaccine Preventable Diseases

### Cholera

Cholera is an acute diarrheal illness caused by ingesting water or food contaminated with the infectious agent, *Vibrio cholerae*. Just like other infectious diseases, cholera can easily spread in areas of poor sanitation with contaminated water supplies such as refugee camps. There have been seven cholera pandemics since the seventeenth century, and most outbreaks have been closely linked to conflict and economic crises (7). It is estimated that 2.9 million cases and 95,000 deaths in endemic countries are annually attributed to cholera (8). In the EMR, 14 out of the 22 countries have witnessed cholera breakouts in the past decade alone (9). Although most people remain asymptomatic or experience mild symptoms, cholera can be deadly, particularly if those afflicted are left without adequate hydration. While it is not routinely administered to children worldwide, the oral cholera vaccine (OCV) may be beneficial in endemic countries where access to proper health care may prove difficult (10).

### Polio

Polio belongs to the *Enterovirus* genus of the *Picornaviridae* family. The virus causes a disease that has long been known to have devastating effects on the children it inflicts, affecting the nervous system and causing partial or total paralysis. By the 1980s, polio was responsible for paralyzing more than one thousand children every day worldwide (11). This prompted the Global Polio Eradication Initiative (GPEI) to be established in 1988, which succeeded in immunizing more than 2.5 billion children against the virus and decreasing the incidence of polio worldwide by 99% since its inception (11). To date, the EMR has seen a total of 10 cases since the start of 2017, a substantial drop from the 74 cases of 2015 (12). Despite the tremendous strides achieved in eradicating this disease, polio continues to pose a significant health problem in affected countries.

### Measles

Another vaccine-preventable disease that has flourished during the chaos of war is measles. The etiological agent is the Measles virus (MeV): a member of the *Paramyxoviridae* family. Measles is a highly contagious airborne disease, which since the advent of a successful vaccine, has largely been eradicated in many parts of the world. The WHO estimates that measles alone was responsible for approximately 9.7 million cases and 254,928 deaths in 2016, with a large proportion of these fatalities occurring among children in developing countries (13). In 1997, all countries of the EMR set measles elimination as a goal to be achieved by 2010, and a four-plan strategy detailing vaccination activities was devised. By 2010, the number of reported measles cases in the EMR decreased by 77% from 1998 (14). With war and conflict taking place in many of these countries, efforts to contain the disease have not always proved successful. Although no specific treatment exists, most relief efforts are aimed toward effective immunization and prevention.

## EMR Countries with Re-Emerging VPDs

### Iraq

In the past three decades, Iraq has undergone many political calamities, leaving the country into a constant state of insecurity. In 2003, the country was thrown into political upheaval that was compounded with United States involvement. Although the Iraq War officially ended in 2011, conflict has continued to involve different religious and ethnic factions, contributing to a crackdown in social order with almost 3 million people internally displaced by 2017 (15). Throughout this, one aspect that has suffered tremendously is the Iraqi health care system.

Prior to the eruption of the Gulf Wars in the 1980s, Iraq provided free health care to its people in 172 hospitals and 1,200 primary health care centers located throughout the country (16). Since then, continuous political and economic instability has left the country with a health care infrastructure that is unable to meet even the most basic health needs of its people. A study performed in Iraq revealed that in 2011, there were only 7.5 physicians for every 10,000 citizens, which is almost half the global ratio of 14/10,000 (17). Another measure of this deterioration is a decline in health indicators, such as life expectancy at birth, which in 2010 was 58 years, a decrease from 65 years in 1980 (18).

In 2000, about 61% of Iraqi children were fully vaccinated (19). Just 1 year after the start of the war in 2004, Iraq witnessed a tremendous surge in the rates of vaccine-preventable diseases. In the first few months after the US invasion, numerous primary health care centers and hospitals were destroyed, vaccinations became short in supply, and access to unvaccinated people in remote areas became difficult.

During the 1980s, polio immunization was mandatory in the country. In the 1990s and until 2003, national polio vaccination campaigns were held annually, and vaccination rates reached those similar to the pre-Gulf War era (19). After 14 years of being polio free, in 2013 the country confirmed its first case of polio in an unvaccinated infant living on the outskirts of Baghdad (20). This was most likely linked to the re-emergence of the virus in neighboring, war-torn Syria. Despite this, and several other cases, since April 2014 there have been no new cases of polio in the country, and Iraq was removed from the list of polio-infected countries in March of the following year (21). In the spring of 2016, a 5-day polio vaccination campaign was set out to vaccinate more than 5 million children under the age of five. The campaign, which was brought about by the Iraqi Ministry of Health, with support from the WHO and UNICEF aimed at preventing a resurgence of the disease (21).

In the early 1980s, Iraq adopted the WHO Expanded Program on Immunization (EPI) leading to a decrease in the annual number of measles cases from 39,000 to 9,400 (22). The country’s immunization schedule included two doses of the measles containing vaccine (MCV1 and MCV2). However, with the advent of political instability in the region, the number increased nearly twofold by 2013 (14). In the first half of 2004, there were 8,253 cases of measles reported in Iraq, which was a significant increase from the 454 cases described in 2003 (23). Vaccination rates plummeted from 87% in 2000 to 69% in 2009. By early 2014, around 1,040 cases had been described in the country, prompting a nation-wide vaccination campaign (24).

Adequate vaccination is crucial to achieve herd immunity and prevent the occurrence of outbreaks. A study conducted in 2012 showed that even those who had received one dose of the MCV vaccine had a 3.7 times lower risk of contracting measles compared to those who had never received it (25). Some of the obstacles impeding the progress of nation-wide vaccination campaigns include mass displacement and migration, inefficient vaccination initiatives, inadequate surveillance, and lack of funds (14).

### South Sudan

After decades of civil war in Sudan, South Sudan finally seceded and declared its independence in 2011, making it the world’s youngest nation. Although the separation was followed by a period of peace, by 2013 civil unrest resurfaced among different ethnic and political factions, eventually leading to one of the worst refugee crises of the modern world. By 2017, of the nearly 13 million South Sudanese citizens, almost 2 million people have been internally displaced, and with another 1.4 million refugees in neighboring countries, mainly Uganda and Ethiopia (26). In addition to on-going violence and civil unrest, the citizens of South Sudan have been subject to rising poverty, malnutrition, and unpredictable natural disasters (27).

Prior to the eruption of war, Sudan employed a government-run Public Health System that was the only provider of health services. After 2011, a three-layered health system structure was adopted consisting of Primary Health Care Units (PHCU), Primary Health Care Centers (PHCC), and hospitals (28). In 2006, following a period of peace in the country, the Government of Southern Sudan formulated the “Basic Package of Health and Nutrition Services” that detailed a plan for immunization program development. The EPI then prepared the “2007–2011 Comprehensive Multi-Year Plan (cMYP) for Immunization” to be used until the country gained independence (29, 30), with a follow-up program designed after few years.

With the rising refugee and migration crisis in the country, the health care system has suffered major setbacks. Currently, the country has one of the worst health indicators worldwide, with life expectancy at the age of 54 years. The health care system is also suffering from a severe shortage of health professionals with one physician for every 65,574 people (31). Despite the many free services offered by the Public Healthcare System, the majority of the population live in impoverished, remote areas with little access to health care facilities (32, 33). This, paired with a government that in 2014 spent only 2.7% of the country’s GDP on health care, has only served to worsen the problem (34).

One recent survey found that the percentage of fully immunized children was only 7.3%, with the percentage of partially immunized children not much higher (35). In 2000, the EPI estimated that measles vaccination coverage was about 63% in the country (36). Although Southern Sudan had endured multiple measles outbreaks in recent decades, with the most notable one occurring in October 2003 (37), by the spring of 2015, the country had witnessed one of the worst measles outbreaks of the region, with a total of 1,730 confirmed cases and 22 deaths. By May 2017, 664 suspected cases and three fatalities were reported (38). This prompted the Ministry of Health, in partnership with UNICEF, to launch a massive vaccination campaign to immunize 7.9 million children between the ages of 6 months and 15 years (39).

Another disease that has flourished in South Sudan in recent years is cholera. Although endemic to the country, a devastating outbreak of the disease has claimed the lives of 320 people and afflicted 17,785 others since June 2016 (40). When it first appeared in 2014, the South Sudanese Ministry of Health, in partnership with WHO, carried out a mass cholera vaccination campaign between February and April 2014, which succeeded in immunizing 60–85% of the target population. A retrospective study found that those who had received two doses of the vaccine were 4.5 times less likely to develop severe disease compared to their unimmunized counterparts (41). However, with a population on the brink of starvation, and often fleeing from areas of conflict, cholera continues to be a deadly disease. By 2015, the global stockpile of the cholera vaccine contained only 900,000 vaccines, making its allocation to the 1 million people at risk an even more difficult task (42).

Despite their successes, vaccination campaigns are not sufficient to solve the growing problem. Access to health care services is often impeded and the presence of refugees and displaced people in rural areas, away from major cities serves only to compound the problem. In addition, an on-going famine in the country has left almost a million people on the brink of starvation, leaving those inflicted immunocompromised and unable to fight off an otherwise mild infection (43).

Similarly, the chaos created by wars and conflict make proper surveillance and monitoring systems of vaccination programs a difficult, yet necessary task. In June 2017, the South Sudanese government announced the death of 15 children due to contaminated measles vaccines (44). Inadequate storage, including lack of refrigeration, and poor administration practices, such as reusing syringes, were cited as some of the reasons for this incident. Any efforts aimed at containing the rise of vaccine preventable diseases in South Sudan needs to tackle the staggering number of health concerns from all aspects.

### Syria

During the 1960s, Syria underwent major political upheaval with the establishment of a socialist government. Despite the political reforms, the country enjoyed relative economic stability, which reflected well on the health care system. During that time, health care primarily consisted of a government-run system that provided citizens with access to nationwide primary health care services (45). Political stability was accompanied by an improvement in overall life expectancy, which rose from 56 years in 1970 to 73.1 years in 2009, and infant mortality decreased from 132 deaths for every 1,000 live births in 1970 to 17.9 per 1,000 in 2009 (46). The country also boasted of a thriving pharmaceutical industry; one that was able to cover almost 90% of the countries national needs, in terms of both local use and international export (47).

Since 2011, the country has been at the center of a political crisis that has taken the world by storm. More than 5 years after the start of the Syrian Civil War, the world has witnessed one of the worst refugee crises seen since WWII (48). A recent WHO survey updated in February 2017 revealed that more than 7.5 million people are displaced within Syria and almost 5 million have fled to neighboring countries such as Lebanon, Jordan, and Turkey (49).

Other than the direct casualties of war, the Syrian crisis has claimed many victims from basic health care issues that otherwise could have been prevented. Hospitals and ambulances were the frequent targets of missiles and explosions, leading to a crumbling of any health care infrastructure previously present. More than 700 medical workers have been killed since the start of the war in 2011 and more than 60 health care facilities have been destroyed in the past year alone (50). Internally displaced people and refugees who have moved to neighboring countries are forced to reside in poor living conditions, with limited access to sanitation and basic health care services. Diseases such as measles and polio, that were once thought eradicated, now found the perfect breeding ground to thrive and spread.

In Syria, prior to the outbreak of the war, the last documented case of polio was reported in 1999. The lack of proper immunization, coupled with contaminated living conditions, led to the re-emergence of polio after many years of obscurity, with the first case documented in October 2013, and eventually reaching 35 cases within the same year (51). The wild-type poliovirus (WPV1) discovered in Syria in the midst of the war is similar to the strain of poliovirus in Pakistan, one of the few countries in the world where polio remains endemic (51). It is believed that the virus found its way to the country by way of Pakistani freedom fighters (52). Prior to the conflict, an estimated 91% of Syrian children had been vaccinated against polio, with that number dropping to 68% in 2012 (51). After decades of eradication, polio once again became a health concern to the Syrian people.

By 2014, the WHO announced that polio had become a Public Health Emergency of International Concern (53). These developments led the GPEI to put forth a multi-country strategy in the region aimed at curbing the spread of the disease. By April 2014, 30 supplementary immunization activities (SIAs) succeeded in reaching more than 25 million children under the age of 5 in war-torn countries like Syria and Iraq, and neighboring countries hosting refugees, such as Jordan and Lebanon (54). This initiative, along with other vaccination campaigns by organizations such as the WHO, has led to a 3-year polio-free period in Syria since 2014 (55). To ensure that recurrence does not happen, the WHO undertook a successful national polio campaign in most Syrian governorates for 5 days in October 2016 reaching a target of 2.8 million children under the age of 5, 83% of the target population (56). These successful efforts were recognized in the 2015 Annual Report of the Polio Global Eradication Initiative that stated that the world is the closest it has ever been to being polio free (57).

Factors that may jeopardize this success include continued conflict, inability to reach besieged areas, and the rapidity by which the disease can spread in poor living conditions. Continued efforts need to be made by both international and local groups to ensure continued immunizations against devastating diseases such as polio. In 2010, a case of polio reported in the Republic of Congo revealed a mutated virus in a previously immunized individual. While there have been no reports in Syria of mutated polio virus infections in vaccinated individuals, it is imperative to keep in mind that effective eradication of the disease will need to take into account possible antigenetic variants that can continue to persist despite adequate vaccination measures (58).

### Yemen

In March 2015, Yemen underwent major political upheaval when war broke out between different religious factions. Since then, thousands have been killed and many more have been displaced. Yemen, which had a fragile health care system prior to the onset of war, did not have the infrastructure to withstand these changes and is currently on the brink of collapse. Prior to the war, Yemen had one of the highest rates of childhood malnutrition worldwide (59). In the past 2 years, continuous airstrikes have led to the destruction of health care facilities and have made access to the ones standing difficult for those in need. Similar to the situation in Iraq, South Sudan, and Syria, vaccine-preventable diseases saw a sudden surge after the beginning of the crisis (60).

Prior to the war, Yemen had a relatively stable vaccination rate reaching 70–80% of the target population (61). By November 2015, measles vaccination dropped to 54% from 75% of the previous year (62). By April 2016, a large house-to-house vaccination campaign undertaken by the Ministry of Public Health and Population in collaboration with the WHO and UNICEF managed to successfully vaccinate more than 90% of targeted children (63). To ensure its continued success, further vaccination campaigns will need to be undertaken in the near future.

On October 6, 2016, health authorities in Yemen announced an outbreak of cholera. By the end of December of the same year, there had been 163 confirmed cases, with 96 associated deaths (64). By June 2017, the United Nations announced that country is currently facing the “worst cholera outbreak in the world” (65), and on August 13th of the same year, the total number of suspected cholera cases reached half a million (66). In June 2017, the International Coordinating Group (ICG) on vaccine provision announced a plan to send half of the global stockpile of cholera vaccine to Yemen in an effort to curb the rise of this devastating infection (67). A month later, the WHO sent hundreds of tons of aid including medical supplies. Other relief organizations, such as the Red Cross and the European Commission, have also contributed similarly (67).

Vulnerable groups include children and the elderly, in addition to immunosuppressed people and those with multiple comorbidities. A failing sewage system, continued conflict and inadequate health care facilities are only a few of the reasons contributing to this problem. Malnutrition, which is a significant consequence of the Yemen War, has further contributed to this outbreak. Without adequate assistance from both local and international relief agencies, cholera is expected to claim many more lives.

## Recommendations for the Future

In 2013, the WHO developed a document titled “Vaccination in acute humanitarian emergencies: a framework for decision making” aimed at helping aid agencies and policy makers with a systematic method of vaccinating in times of humanitarian crises, that is developed to take epidemiological and contextual factors into account (68). The WHO and various United Nations agencies have already undergone successful vaccination campaigns in warring countries of the EMR, and in neighboring countries hosting refugees. Since the implementation of vaccination campaigns during active warfare is dangerous for all those involved, National Immunization Days (NID) have long been used as a way of negotiating truces or ceasefires. To ensure continued success, follow-up campaigns are needed, particularly for diseases that require booster immunizations. In cases of refugees living in nearby countries, it is essential to work with local governments and organizations to ensure concerted efforts of immunization. Vaccination efforts should include reaching remote regions, and not just people located in major cities.

Adequate monitoring of vaccination, through the use of EPI cards and other validated recall methods, is crucial to ensure adequate surveillance of vaccination rates and coverage (69). Appropriate transportation and storage of vaccines, particularly in difficult-to-access regions, are also needed to ensure continued adequate coverage. Similarly, in order to prevent another shortage of vaccines, such as the one seen in South Sudan in 2015, the global stockpile needs to be fully equipped and capable of providing vaccines quickly during emergency situations and to large numbers of people (42).

Delayed identification and treatment contribute to the spread of infectious diseases, making them even harder to control. Health care volunteers may unknowingly become carriers of diseases and transmit them back to their home countries. With globalization and the advent of international travel, diseases that were once restricted to a particular community now threaten to inflict communities worldwide (70). Speedy and accurate diagnosis is also needed to mobilize relief activities quickly and effectively.

While immediate medical attention is in dire need, there is a need to re-focus efforts toward long-term public health measures that are likely to benefit people for years to come. Post-conflict Needs Assessments (PCNAs), which are multilateral exercises needed to evaluate the health care services in affected areas, should be undertaken by international organizations in collaboration with national governments (71). Financial strains in the form of operational costs are another barrier impeding adequate health care, and relief organizations and foreign money are not a sustainable solution for the long run (72).

Many health facilities in the aforementioned countries have been destroyed by conflict, and the lack of the electricity and running water makes the operation of any health care infrastructure difficult to achieve. Security concerns prevent volunteer workers and patients from venturing out of safe zones in search of health services. Wars also often drive highly educated professionals such as nurses and doctors to flee, leaving the country with a shortage of medical workers that are unable to shoulder the burden of a large, ailing population. Medical equipment and facilities are as scarce as those that operate them. Any future talks about re-building these countries’ health care systems need to include sustainable investments in infrastructure.

In addition to infrastructure, it is important that health care providers choose appropriate methods for approaching those in need of medical care. For example, refugees have been shown to be less likely than others to seek health care. The costs of vaccination were cited as a major barrier for seeking health care among Syrian refugees in Jordan (73). Also, language barriers, discrimination and legal obstacles may deter some from reaching out to health care facilities (74). Adjusting to a new culture, compounded with increased stress and anxiety have also been cited as some reasons that refugees avoid seeking health care (75). Public education campaigns, using the appropriate languages and dialects, are also needed to motivate people to seek immunization.

## Conclusion

Polio, measles, and cholera are but a few of the re-emerging vaccine preventable diseases that continue to pose a significant health burden in developing countries and in regions of political instability. While reports of war tend to focus on the casualties caused by airstrikes and ammunition, the long-term health consequences caused by inadequate prevention and treatment of communicable diseases are often ignored. War-affected countries rely on the efforts of the international community to recognize that the re-emergence of vaccine-preventable diseases is a global threat that requires a multinational approach. Until the political situations are resolved in these war-torn countries, infectious diseases will continue to spread and inflict vulnerable populations. We are now more than a decade into the twenty-first century, and re-emerging infectious diseases in times of war continue to pose a real and frightening threat to mankind.

## Ethics Statement

As no animals or humans were involved in this research endeavor, institutional review board approval was not required.

## Author Contributions

IH provided the idea and supervised the whole process from inception to the final editing and submission of the manuscript. RR is the first author and did the literature search and wrote the whole manuscript. SS, NC, SC, and AL did the literature, drafted the paper, and critically revised it for important intellectual content. All authors gave final approval of the version to be published.

## Conflict of Interest Statement

The authors declare that the research was conducted in the absence of any commercial or financial relationships that could be construed as a potential conflict of interest.
